# Novel nonsense mutation p. Gln264Ter in the *ANK1* confirms causative role for hereditary spherocytosis: a case report

**DOI:** 10.1186/s12881-020-01161-4

**Published:** 2020-11-13

**Authors:** Senmao Chai, Rong Jiao, Xiaodong Sun, Pan Fu, Qiang Zhao, Ming Sang

**Affiliations:** 1 Hubei Institute of Parkinson’s Disease at Xiangyang No.1 People’s Hospital, Hubei Key Laboratory of Wudang Local Chinese Medicine Research, Hubei University of Medicine, Shiyan, 442000 People’s Republic of China; 2 Department of New pediatric, Xiangyang No.1 People’s Hospital, Hubei University of Medicine, Xiangyang, Hubei China

**Keywords:** Hereditary spherocytosis, Whole-exome sequencing, *ANK1*, Nonsense mutation

## Abstract

**Background:**

Hereditary spherocytosis (HS) is the most common haemolytic anaemia caused by congenital membrane defects of red blood cells. The name derives from the presence of spherical red blood cells in the peripheral blood. Clinical manifestations of HS are anaemia, haemolytic jaundice, and large spleen, and infection can worsen the condition, often with cholelithiasis. HS is mainly caused by abnormal functions of the products of six genes. Splenectomy is the main treatment for HS.

**Case presentation:**

Half a day after birth, the proband exhibited HS-related symptoms, with progressive aggravation. Routine examination in the outpatient department showed an increase in white blood cells and a decrease in red blood cells. His mother had HS and a partial splenectomy. We suspected that the infant might also have HS. Genomic DNA samples were extracted from the three members of the HS trio pedigree, and genomic whole-exome sequencing (WES) was performed. The three DNA samples were amplified by polymerase chain reaction (PCR), followed by Sanger sequencing to identify mutation sites. A novel nonsense heterozygous mutation, c.790C > T (p. Gln264Ter), in the *ANK1* gene, which causes premature termination of translation, was found in this Chinese family with autosomal dominant HS.

**Conclusions:**

This de novo nonsense mutation can cause the onset of HS in early childhood, with severe symptoms. Expanding the *ANK1* genotype mutation spectrum will lay a foundation for the further application of mutation screening in genetic counselling.

## Background

Hereditary spherocytosis (HS, OMIM:182900), also known as congenital hemolytic anemia, refers to one type of heterogeneous inherited anaemia characterized by the presence of spherocytes on the peripheral blood smear [1]. HS occurs all over the world, affecting approximately 1/5000 people in Europe and North America [2, 3]. The main result is defects in the erythrocyte membrane skeleton, resulting in decreased surface area and spherical changes; these changes lead to loss of erythrocyte membrane elasticity and mechanical stability and difficulty in passing through the splenic capillaries, promoting erythrocyte clearance by the spleen [4]. The increase in red blood cell destruction leads to increases in bilirubin levels and causes yellowing of the skin and sclera. The spleen also grows larger due to the destruction of red blood cells and the stimulation of proliferation, followed by liver enlargement. Therefore, anaemia, jaundice, large liver and large spleen are the main symptoms of HS. Currently, the main clinical treatment is partial or total splenectomy and splenic artery embolization. Theoretically, splenic circulation is not fully developed until after birth, resulting in enhanced destruction of the globular cells surrounding the circulation and increased haemolysis. Occasional visits to the hospital cause a high rate of misdiagnosis and delayed treatment, which threatens the life of patients with HS [4]. At present, 6 abnormal genes which lead to HS have been identified. They are *ANK1*(ankyrin; MIM: 182900), *SPTB* (beta-spectrin; MIM: 616649), *SPTA1*(alpha-spectrin; MIM: 270970), *SLC4A1*(Band 3; MIM; 612,653) *EPB41*(protein 4.1; Phenotype MIM:611804) and *EPB42*(protein 4.2;MIM: 612690) [5]. There exist two genetic inheritance patterns for HS: autosomal dominant (AD) (75%) and autosomal recessive (AR) (25%) [2]. HS-causative mutations in *SPTB* and *SLC4A1* show AD, whereas those in *SPTA1*, *EPB41*and *EPB42* show AR; alterations in *ANK1* display AD or AR [2]. Although splenectomy greatly helps patients with HS, it also produces many complications. Some patients even die of infection of mesentery or portal vein occlusion after splenectomy. The most common complication is infection, especially among infants and young children.

In this study, we collected a trio pedigree of HS. The proband showed HS-related symptoms half a day after birth. Through gene sequencing, we identified a de novo nonsense variant in *ANK1* in this family, promoting further application of mutation screening in genetic counselling.

## Case presentation

### Ethical compliance and human subjects

The study was approved by the Ethics Committee of Xiangyang No.1 People’s Hospital, Hubei. Informed consent was obtained directly from the participants or parents/legal guardians of any participant under the age of 18. Blood specimens were collected from the proband and his parents. The proband was a male infant born at full term with an uneventful pregnancy. Apgar scores were 9/1 min and 10/5 min. The proband showed HS-related symptoms with gradual aggravation within half a day after birth. Routine outpatient department examination showed an increased white blood cell count and a decreased red blood cell count. The proband’s mother had a history of HS and underwent partial splenectomy. We suspected the son might have HS. The probability of triggering haemolysis and entire haemolysis is increased in patients with erythrocyte brittleness (Table 1). Based on differential diagnosis, physiological jaundice, neonatal ABO haemolysis, neonatal sepsis, neonatal hepatitis syndrome and bilirubin encephalopathy were all excluded. Amoxicillin and clavulanate potassium were given for anti-infection treatment. The patient was treated with hydration, phototherapy, sodium bicarbonate, and vitamin K1 supplementation. Neonatal diseases are treated in the intensive care unit (ICU) of our hospital, with mother-infant separation.

**Table 1:** Abnormal examination results on admission

Test item	Result	Cue	reference range
Erythrocyte	3.26 × 10^12^/L	↓	(5.20–6.40) × 10^12^/L
Hemoglobin	112.0 g/L	↓	180.0–190.0 g/L
Neutrophils percentage	79.2%	↑	31.0–40.0%
Lymphocyte percentage	13.6%	↓	40.0–60.0%
Red cell distribution width	19.0%	↑	11.5–14.5%
Red cell distribution width standard deviation	66.7 fL	↑	35.0–56.0 fL
Began hemolysis in patients with erythrocyte brittleness	5.40 g/L	↑	3.8–4.6 g/L
Entirely hemolysis in patients with erythrocyte brittleness	4.32 g/L	↑	2.8–3.2 g/L
Total bilirubin	194.33 μmol/L	↑	0.0–26.0 μmol/L
Direct bilirubin	12.47 μmol/L	↑	0.0–8.0 μmol/L
Indirect bilirubin	181.86 μmol/L	↑	0.0-18 μmol/L
γ-Glutamyl transferase	84.38 IU/L	↑	10–60 IU/L
Alkaline phosphatase	129.81 IU/L	↑	45-125 IU/L
Creatine kinase	820 U/L	↑	50-310 U/L
Creatine kinase MB isoenzyme	44.48 U/L	↑	0-24 U/L
Lactic dehydrogenase	385 U/L	↑	120-250 U/L
α- Hydroxybutyrate Dehydrogenase	281.7 U/L	↑	72-182 U/L
C- Reactive Protein	10.75 mg/L	↑	0.0-8 mg/L

### Whole-exome sequencing (WES) and sanger sequencing validation

**Fig. 1:**
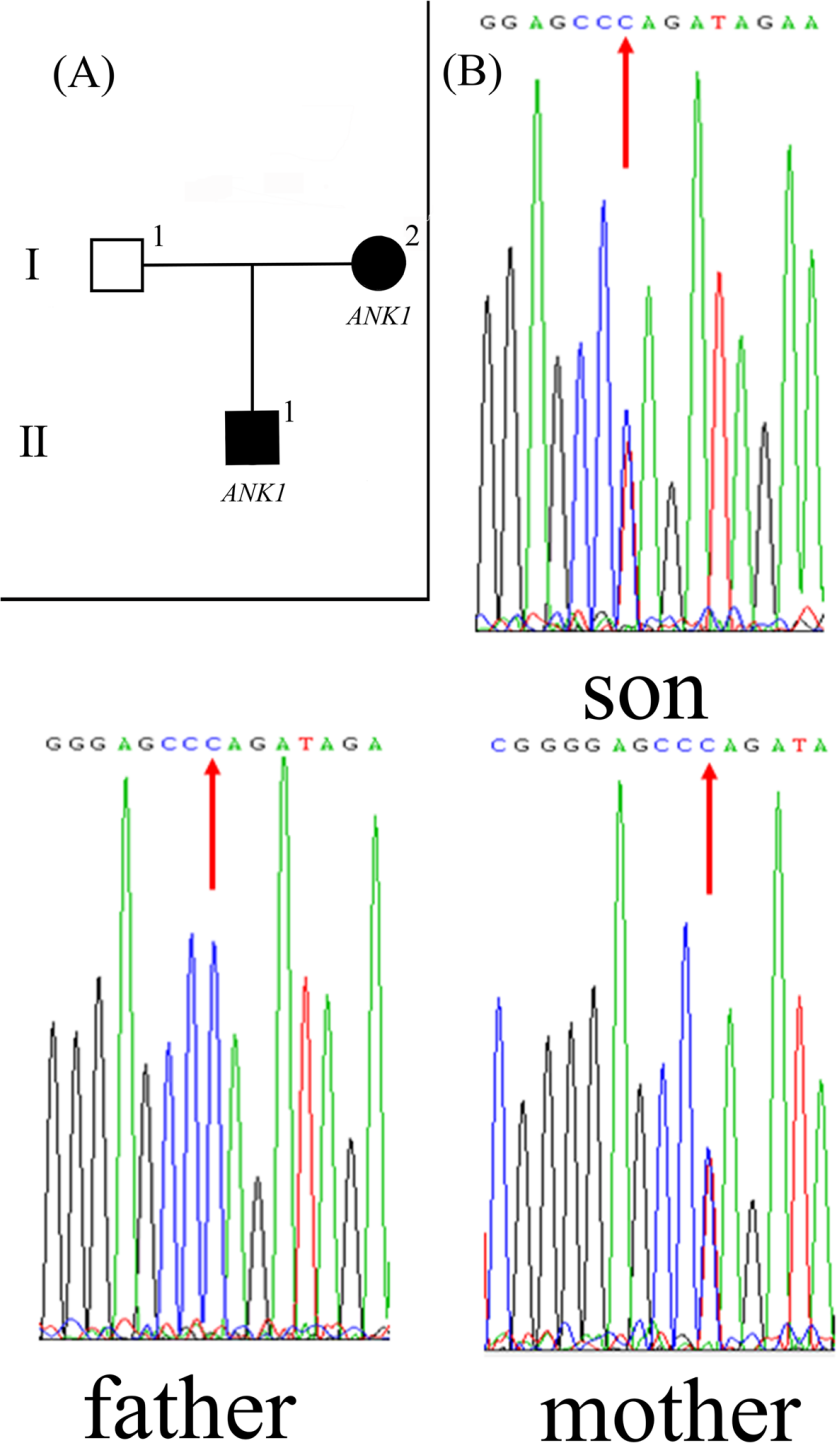
The novel pathogenic variant in the *ANK1* gene. **a** The family tree. **b** The hemizygous variant confirmed by Sanger sequencing. The heterozygous mutations has been carried by Mother and son. The red arrow indicates the mutation site

Genomic DNA samples from each member of the HS trio pedigree (II:1, in Fig. 1a) were extracted from whole blood and used to sequence the whole exome. The BGI Exome V4 kit (BGI) was used for in-solution enrichment of coding exons and flanking intronic sequences, as per the manufacturer’s standard protocol. We used cutadapt (v1.15) to trim adaptor sequences at the tail of the sequencing reads and then aligned the sequencing reads to the human reference genome (UCSC hg19) with BWA (v0.7.15). Duplicated reads marked by Picard (v2.4.1). Qualimap [6] (v2.2.1) was used to calculate base quality metrics, the genome mapping rate, and the coverage of targeted regions. Base quality score recalibration, indel realignment and variant (SNV & InDel) calling were performed following the best practice protocol of Genome Analysis Toolkit (GATK, v3.8). Variant filtering was performed by a finely tuned in-house script. Pass-filter variants were annotated by the Pubvar variant annotation engine (www.pubvar.com) and VEP [7]. Variant models for dominant inheritance and recessive inheritance were separately identified in the genetic analysis. Variants that met any of the following criteria were excluded from the analysis: maximum population frequency larger than 0.01, low genotype confidence, or predicted as benign by all five algorithms, i.e., SIFT [8], PolyPhen 2 [9], MetaSVM [10], MCAP [11] and MutationTaster [12]. The pathogenic evidences of candidate disease-causing variants were scored by InterVar [13] (1.0.8) according to American College of Medical Genetics and Genomics (ACMG) guidelines [14]. Subsequently, all variants in previously reported HS genes (*ANK1*, *SPTB*, *SPTA1*, *SLC4A1 EPB41* and *EPB42*) were investigated for segregation. Finally, all variants following autosomal dominant and autosomal recessive inheritance were checked for segregation.

A novel *ANK1* (NM_000037.3: c.790C > T, p. Gln264Ter) nonsense heterozygous mutation was identified in the infant and his mother. According to the evidence classification of American College of Medical Genetics and Genomics (ACMG) pathogenicity, the mutation was judged to be pathogenic (PVS1 + PM2 + PP4). Frequency data in 1000 Genomes, gnomAD exomes and East Asian gnomAD exomes were not found for this *ANK1* mutation site.

Primer-BLAST (https://www.ncbi.nlm.nih.gov/tools/primer-blast/) was used to design primers containing the mutation site: forward CAGAAATGCCCTGACAACGC and reverse ACTATTGCCTCCCACTGCAC. The annealing temperature was 60 °C, and the amplified fragment size was 395 bp. To verify the *ANK1* mutation, PCR followed by Sanger sequencing were performed for the three individuals (I-1, I-2, II-1) of the pedigree. Our results confirmed that a heterozygous mutation was carried by the mother (I-2) and the son (II-1, Table 2, Fig. 1b). This *ANK1* nonsense heterozygous mutation (NM_000037.3: c.790C > T, p. Gln264Ter) is novel and not reported or listed in the Human Gene Mutation Database (HGMD), Exome Variant Server (EVS), the 1000 Genomes databases or any other database (gnomAD, ExAC, ClinVar, dbSNP). Taken together, the causative mutation was verified by examining the trio pedigree via WES and Sanger sequencing.

**Table 2:** *ANK1* mutation of the family members

Family members	Exon	Reference sequence	Type	cDNA	Protein
**Father**	8	NM_000037.3	Wild	c.790C	p. Gln264
**Mather**	8	NM_000037.3	Mutant	c.790C > T	p. Gln264Ter
**propositus**	8	NM_000037.3	Mutant	c.790C > T	p. Gln264Ter

## Discussion and conclusion

**Fig. 2:**
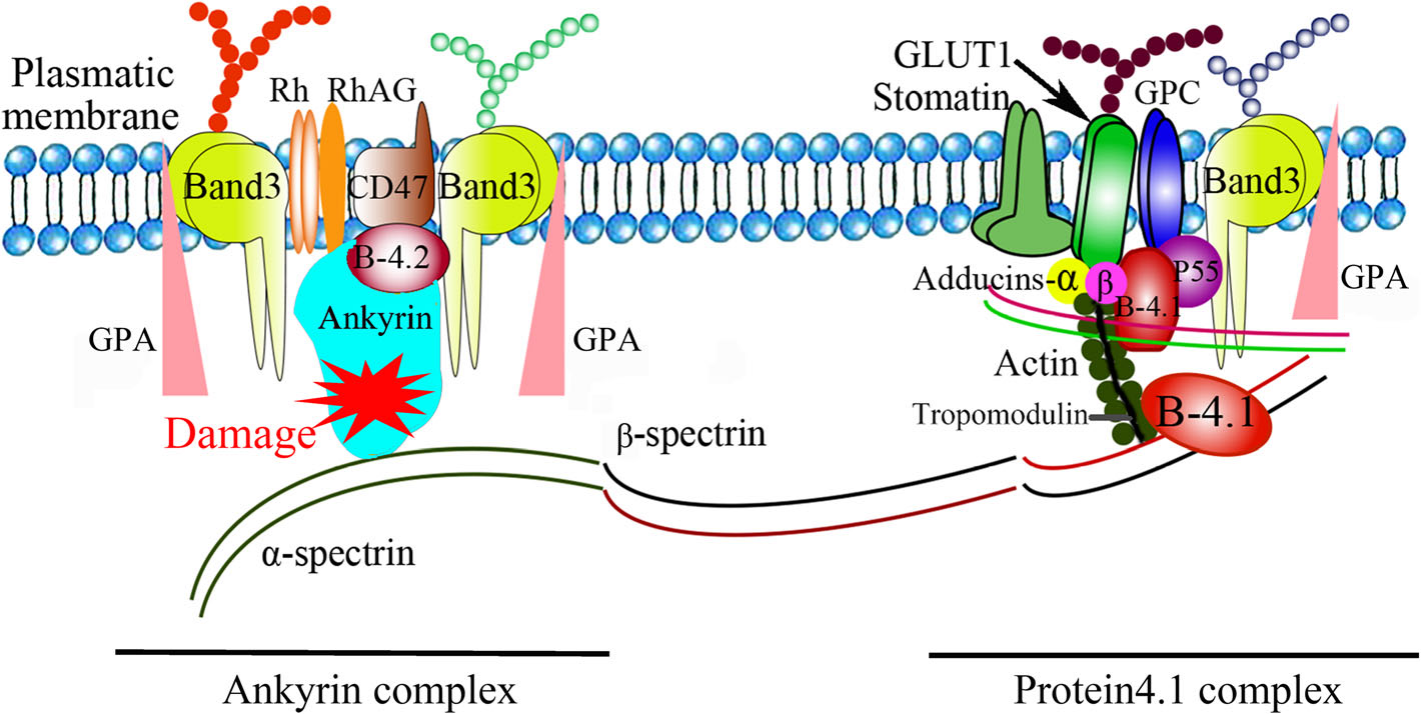
A simplified cross section diagram of erythrocyte membrane. Phospholipid bilayer is the basic scaffold of red blood cell membrane. Protein Ankyrin is marked in red to indicate damage due to gene mutation. At present, it has been found that 6 proteins Ankyrin; Spectrin alpha-chain; Spectrin beta-chain; Band 3; Protein 4.1 and Protein 4.2 paly a pathogenic role in Hereditary spherocytosis. The relative positions of proteins in various complexes are mostly unknown. The shapes of the major proteins are fictitious. GPA: glycophorin A; Rh: Rhesus polypeptide; B-4.1: protein band 4.1; B-4.2: protein band 4.2; GPC: glycophorin C; RhAG: Rh-associated glycoprotein

The cytoskeleton plays a key role in maintaining the morphology and function of erythrocyte membranes. Many proteins, such as ankyrin, spectrin α- and β-chains, proteins 4.1, or 4.1R and actin, cover the inner surface of the erythrocyte membrane to form two protein complexes, the ankyrin and protein 4.1 complex (Fig. 2). These two complexes interact to maintain the circular pie-like structure of the central fovea of red blood cells. The former complex consists of Band 3 tetramers, Rh, RhAG, CD47, Glycophorin A and Protein 4.2; the latter consists of Band 3 dimers binding Adducins α and β, Glycophorin C, GLUT1 and Stomatin [15, 16]. *ANK1*, the most common of the 6 pathogenic genes of HS, is an important member of the anchoring protein family, which is mainly expressed in the erythrocyte membrane. Anchoring proteins are connected to complete membrane proteins, further connected to the spectrin and cytoskeleton. They play an important role in cell movement, activation and proliferation and participate in maintaining the specific morphology of cell membranes. Mutation of the *ANK1* gene can cause red blood cells to change from a double concave disc shape to a spherical shape and reduce its deforming capacity. In capillary networks such as the liver and spleen, rupture and haemolysis may occur due to mechanical damage [17]. There are more than 60 known mutations in the human HS-related *ANK1* gene, among which the most common include missense mutations, nonsense mutations, frame shifts and splice site mutations. The clinical severity of HS depends on the degree of membrane loss, and most ANK1 mutations show AD inheritance [17, 18]. Ankyrin is the main component of erythrocyte membranes and composed of three structural domains: a 24 homologous repeat N-terminal membrane-binding domain (MBD) involved in the binding of Band 3 protein; a Spectrin-binding central domain (SBD); and a least conserved carboxy-terminal regulatory domain subject to variation [19].

This clinical report shows that the proband and his mother carry a de novo nonsense heterozygous mutation in the *ANK1* (NM_000037.3: c.790C > T, p. Gln264Ter) gene. In contrast to most affected newborns, this proband presented severe anaemia in a very early life and remained transfusion dependent. Half a day after birth, his skin appeared yellow dye, and suffered from progressive aggravation, his white blood cell counts and c-reactive protein both were increased significantly. Our findings show the de novo nonsense mutation can cause the onset of the disease in early childhood, even with more severer symptoms. Expanding the genotypic mutation spectrum of *ANK1*, can work for further application of mutation screening in the genetic counseling.

## Data Availability

All relevant data are within the paper, including raw data from WES, Further information is available from the authors on request. All raw data related to our study has been submitted on NCBI Sequence Read Archive (SRA) (SRA RunSelector: https://www.ncbi.nlm.nih.gov/sra/PRJNA671560). Accession number is PRJNA671560. Reference datasets were listed in the “Whole-exome sequencing (WES) and Sanger sequencing validation” section, such as the human reference genome (UCSC hg19) and transcription number (NM_000037.3).

## Abbreviations

HS: Hereditary spherocytosis
AD: Autosomal dominant
AR: Autosomal recessive
*ANK1*: Ankyrin 1
*SPTB*: Beta-spectrin
*SPTA1*: Alpha-spectrin
*SLC4A1*: Band 3
*EPB41*: Protein 4.1
*EPB42*: Protein 4.2
WES: Whole exome sequencing
ACMG: American College of Medical Genetics and Genomics
